# Two new species of the millipede genus *Metonomastus* Attems, 1937 from the Balkan Peninsula (Diplopoda, Polydesmida, Paradoxosomatidae)

**DOI:** 10.3897/zookeys.786.28386

**Published:** 2018-09-25

**Authors:** Dragan Antić, Boyan Vagalinski, Pavel Stoev, Sergei Golovatch

**Affiliations:** 1 Institute of Zoology, University of Belgrade-Faculty of Biology, Studentski Trg 16, 11000 Belgrade, Serbia University of Belgrade Belgrade Serbia; 2 Serbian Biospeleological Society, Trg Dositeja Obradovića 2, 21000 Novi Sad, Serbia Serbian Biospeleological Society Novi Sad Serbia; 3 Institute of Biodiversity and Ecosystem Research at the Bulgarian Academy of Sciences, 2 Yurii Gagarin Street, 1113, Sofia, Bulgaria Institute of Biodiversity and Ecosystem Research at the Bulgarian Academy of Sciences Sofia Bulgaria; 4 National Museum of Natural History, Sofia, Tsar Osvoboditel Blvd. 1, Sofia 1000, Bulgaria National Museum of Natural History Sofia Bulgaria; 5 Pensoft Publishers, Sofia, Bulgaria Pensoft Publishers Sofia Bulgaria; 6 Institute for Problems of Ecology and Evolution, Russian Academy of Sciences, Leninsky pr. 33, Moscow 119071, Russia Institute for Problems of Ecology and Evolution, Russian Academy of Sciences Moscow Russia

**Keywords:** Bulgaria, cave, Croatia, Dinarides, island, millipede, Rhodopes, taxonomy

## Abstract

In addition to the eleven previously known species of the Mediterranean genus *Metonomastus*, two more species are described: *M.petrovi***sp. n.**, from the Rhodopi Mts. and Bunardzhik Hill in Bulgaria, and *M.radjai***sp. n**., from the island of Mljet in Croatia. The relationships between the congeners and their distributions are briefly discussed. All 13 species of the genus are keyed.

## Introduction

In the latest taxonomic survey of the genus *Metonomastus* Attems, 1937, Golovatch and Stoev (2004) recognized eleven species. Among these, only one species, *M.bosniensis* (Verhoeff, 1901), was considered dubious as it had been described from a single female (holotype) coming from within the distribution area of *M.albus* (Verhoeff, 1901), a fairly common and abundant congener. This Mediterranean genus includes only small-sized forms (< 10 mm long) ranging from the Apennine and Balkan peninsulas in the west to northwestern Anatolia in the east (Hoffman and Lohmander 1968; Golovatch and Stoev 2004).

The present paper is devoted to descriptions of two new species of *Metonomastus* found in the Rhodopi Mountains and Bunardzhik Hill in Bulgaria and the Dinaric island of Mljet in Croatia.

## Material and methods

### Preservation, dissecting, imaging, and terminology

Specimens preserved in 70% ethanol were examined with Nikon SMZ 745T and Zeiss Stemi 2000-C binocular stereo microscopes. All taxonomically important structures were dissected and mounted in glycerine as temporary microscopic preparations and observed with a Carl Zeiss Axioscope 40 and an Olympus BX51 light microscope. Pictures of legs were taken with a Canon PowerShot A80 digital camera connected to the Axioscope 40 microscope, and with an Olympus XC30 digital camera connected to the Olympus BX51 microscope. Line drawings of gonopods were made using tracing paper placed on a computer monitor showing pictures of those structures. Pictures of habitus structures were taken using a Nikon DS-Fi2 camera with a Nikon DS-L3 camera controller attached to a Nikon SMZ 1270 binocular stereo microscope, and with a Nikon Coolpix S3700 attached to one eyepiece of a Carl Zeiss Discovery.V8 binocular stereo microscope. Focal stacking was completed with Zerene Stacker software. Some relevant structures were investigated with JEOL JSM-6460LV (University Centre for Electron Microscopy, Department of Biology and Ecology, University of Novi Sad, Serbia) and JEOL JSM-5510 (Faculty of Chemistry, Sofia University) scanning electron microscopes.

Descriptions of the new taxa largely follow Golovatch and Stoev (2004), except for the gonopodal terms “tibiotarsus” and “prefemur” which are replaced by “solenophore” and “prefemorite”, respectively.

The final images were processed with Adobe Photoshop CS6.

Abbreviations used to denote gonopodal structures are explained directly in figure captions.

### Museum and collection acronyms

**IZB** Institute of Zoology, University of Belgrade – Faculty of Biology, Belgrade, Serbia

**NHMSC** Natural History Museum, Split, Croatia

**NMNHS** National Museum of Natural History, Sofia, Bulgaria

## Taxonomy

### Genus Metonomastus

Taxon classificationAnimaliaPolydesmidaParadoxosomatidae

Attems, 1937

Microdesmus Verhoeff, 1901: 223; preoccupied, replaced with Metonomastus by Attems (1937: 46)
Nannodesmus Chamberlin, 1943: 35; replacement name for Microdesmus Verhoeff, 1901; synonymized by Hoffman and Lohmander (1968: 72)
Microdesminus Strasser, 1960: 96; synonymized by Golovatch and Stoev (2004: 203)

#### Diagnosis.

**Figure 1.**
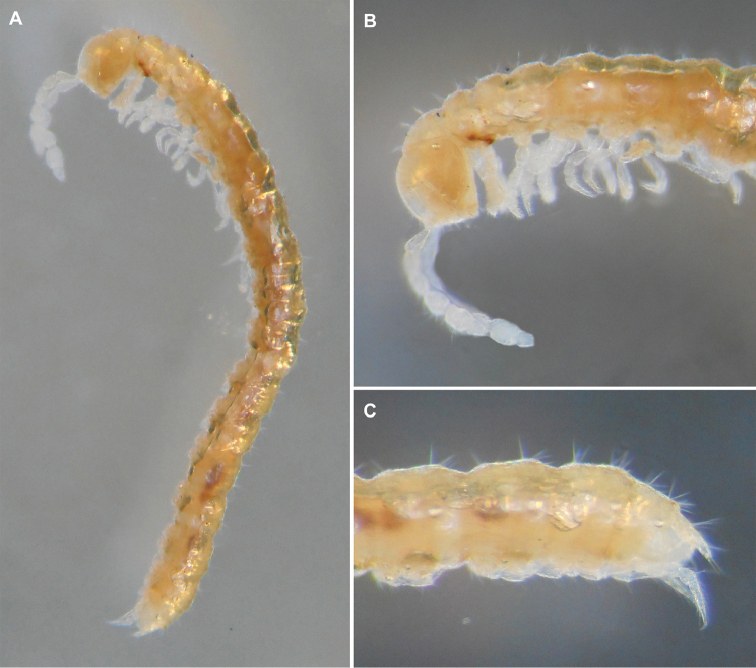
*Metonomastuspetrovi* sp. n., holotype ♂ **A** habitus, lateral view **B** anterior part of the body, lateral view **C** posterior part of the body, lateral view.

Small (< 10 mm long) moniliform polydesmoids with pale body and 19 segments in both sexes (Figs 1, 5). Metaterga mostly with 2–3 rows of setae; exceptions are *M.saetosus* (Strasser, 1960) and *M.bosniensis* (Verhoeff, 1901) (see below). Pleurosternal carinae poorly developed, present only on ring 2 or on rings 2–4. Antennae long, clavate; antennomeres 5 and 6 each with a group of bacilliform sensilla. In males, femora of pregonopodal legs enlarged, each usually with a small adenostyle. Gonopods directed anteriad, with a somewhat elongated and slender coxite (cx). Prefemorite (pf) densely setose, mostly elongated, at least equal in length to acropodite, the only exception is *M.albus* with a shorter prefemorite. Acropodite consisting of two or three, more or less well-developed branches. The two dominant branches are mostly solenomere (sl) and solenophore (sph); the exception is *M.petrovi* sp. n. with three acropodital branches, but with a completely reduced solenophore.

### Included species

*Metonomastusalbus* (Verhoeff, 1901)

*M.bosniensis* (Verhoeff, 1901)

*M.capreae* (Verhoeff, 1942)

*M.hirtellus* (Silvestri, 1903)

*M.mariae* (Strasser, 1965)

*M.patrizii* Manfredi, 1950

*M.petrelensis* Mauriès, Golovatch & Stoev, 1997

*M.petrovi* sp. n.

*M.pomak* Golovatch & Stoev, 2004

*M.radjai* sp. n.

*M.romanus* (Verhoeff, 1951)

*M.saetosus* (Strasser, 1960)

*M.strasseri* Hoffman & Lohmander, 1968

### Metonomastus petrovi sp. n.

Taxon classificationAnimaliaPolydesmidaParadoxosomatidae

http://zoobank.org/ADAE2B3A-37D6-4EB0-B152-5CA394ED5518

Figs 1 , 2 , 3 , 4

#### Material examined.

**Holotype** male (NMNHS), Bulgaria, Western Rhodopi Mts., Satovcha District, Cave Stapalkata, 650 m a.s.l., clay-guano, 17.VI.2006, B. Petrov & P. Stoev leg.

#### Paratypes.

2 males, 5 females (NMNHS), same data as holotype.

#### Additional material.

2 females, 1 juvenile (NMNHS), Bulgaria, Western Rhodopi Mts., Pazardzhik District, Peshtera town, Cave Snezhanka, 19.IV.2009, P. Beron leg.; 1 male, 1 female (NMNHS), Bulgaria, Plovdiv, Bunardzhik Hill, under decaying wood, 10.IV.2018, P. Mitov leg.

#### Etymology.

The species is named after Boyan Petrov, a renowned Bulgarian mountaineer, a dear friend and colleague zoologist from the National Museum of Natural History, Sofia, who disappeared in Tibet in May 2018 during the ascent of his 11^th^ eight-thousander, Shishapangma. Boyan was one of the collectors of this new species. Noun in genitive case.

#### Diagnosis.

The new species belongs to the *Metonomastus* group of species with three acropodital branches, but clearly differs from both previously described species in this group, *M.strasseri* Hoffman & Lohmander, 1968, and *M.pomak* Golovatch & Stoev, 2004, by the presence of a strongly developed, beak-shaped solenomere, by the completely reduced solenophore and by the development of a strongly arched, microspiculate, ventromesal, acropodital process.

#### Description.

Length 4.4–4.8 mm (males), 4.2–5.5 mm (females). Width of midbody rings 0.31–0.37 and 0.33–0.42 mm (males), 0.35–0.42 and 0.42–0.47 mm (females) on pro- and metazonae, respectively. Holotype male 4.8 mm long, 0.37 and 0.42 mm wide on midbody pro- and metazonae, respectively.

**Figure 2.**
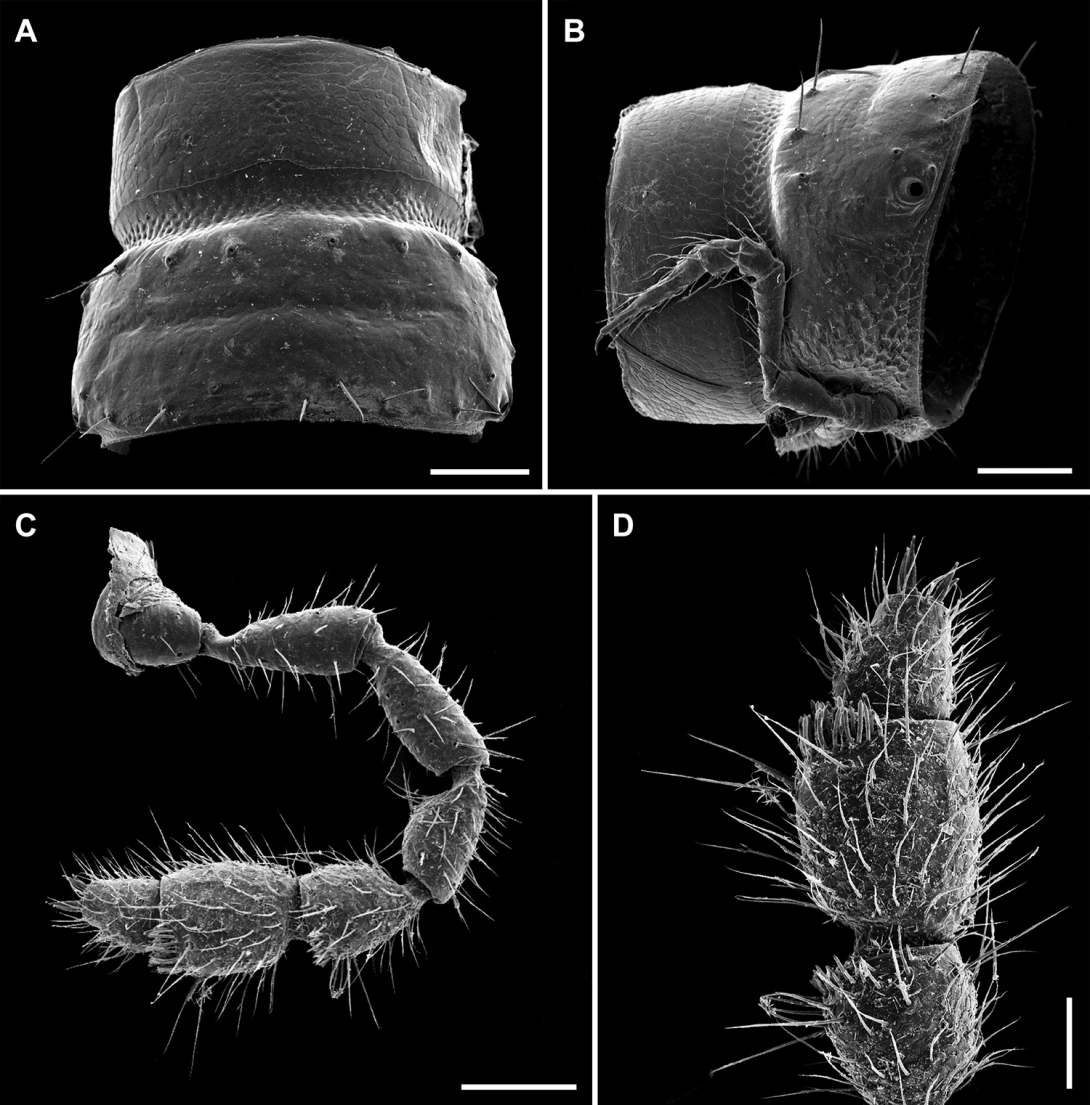
*Metonomastuspetrovi* sp. n. **A** paratype ♀, ring 9, dorsal view **B** paratype ♀, ring 10, lateral view **C** paratype ♂, left antenna **D** paratype ♂, tip of left antenna. Scale bars: 0.1 mm (**A, B, C**), 0.05 mm (**D**).

Body moniliform (Figure 1A), with 19 segments in both sexes. Colouration entirely pallid. Texture microreticulate throughout (Figure 2A, B).

Head densely pubescent throughout, clypeolabral region densely setose. In males, width of head (0.41–0.5 mm broad) >> collum ≤ ring 2 ≤ 3 ≥ 4 < 5 ≥ 6 = 17; thereafter body gradually, but significantly tapering. In females, width of head (0.42–0.5 mm broad) >> collum = ring 2 < 3 = 4 < 5 = 17; thereafter body rather rapidly tapering; in larger females, ring 5 = 14 < 15 = 17.

Antennae (Figure 2C, D) long, clavate, *in situ* reaching behind ring 2 when stretched dorsally. Antennomere length 2 = 3 = 6 > 4 = 5 = 7 > 1, 6^th^ being the thickest, both 6^th^ and 5^th^ each with a compact group of bacilliform sensilla dorso-apically. Interantennal isthmus ca 0.7 times as broad as diameter of antennal socket.

Collum with 3 rows of setae; two rows of similar setae per postcollum metatergum: one frontally, the other caudally, setae long and simple. Metaterga (Figure 2A, B) with a weak, mid-dorsal, transverse sulcus on rings 5 to 17; sulcus absent from 18^th^. Paraterga (Figure 2A, B) laterally extremely poorly developed, being very faintly delimited by a shallow sulcus only dorsally. Certain midbody rings occasionally with visibly more or less strongly developed paranota compared to neighboring rings. Ozopores indistinct, located near posterior margin of paraterga; pore formula normal. Strictures between pro- and metazonae very faintly striolate, deep and narrow. Pleurosternal carinae present on ring 2 as small lobes, thereafter missing. Limbus faintly microcrenulate. Epiproct long, slender, nearly half as long as telson height. Hypoproct semi-circular, 1+1 strongly separated caudal setae borne on minute knobs. Sterna broad and weakly impressed.

**Figure 3.**
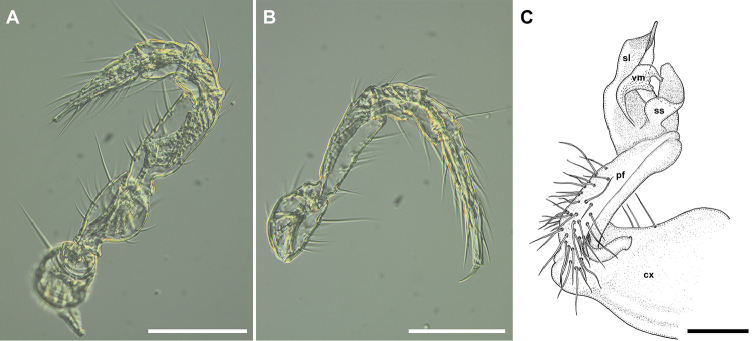
*Metonomastuspetrovi* sp. n., paratype ♂ **A** leg 4 (claw fallen off) **B** leg 6 **C** right gonopod, mesal view. Abbreviations: **cx** coxite, **pf** prefemorite, **sl** solenomere, **ss** saddle-shaped process, **vm** ventromesal process. Scale bars: 0.1 mm (**A, B**), 0.05 mm (**C**).

Legs (Figure 2B) about 1.1–1.2 times as long as midbody height in males, 0.8–0.9 times in females, without modifications, only pregonopodal male prefemora slightly bulged dorsally (Figure 3A, B).

**Figure 4.**
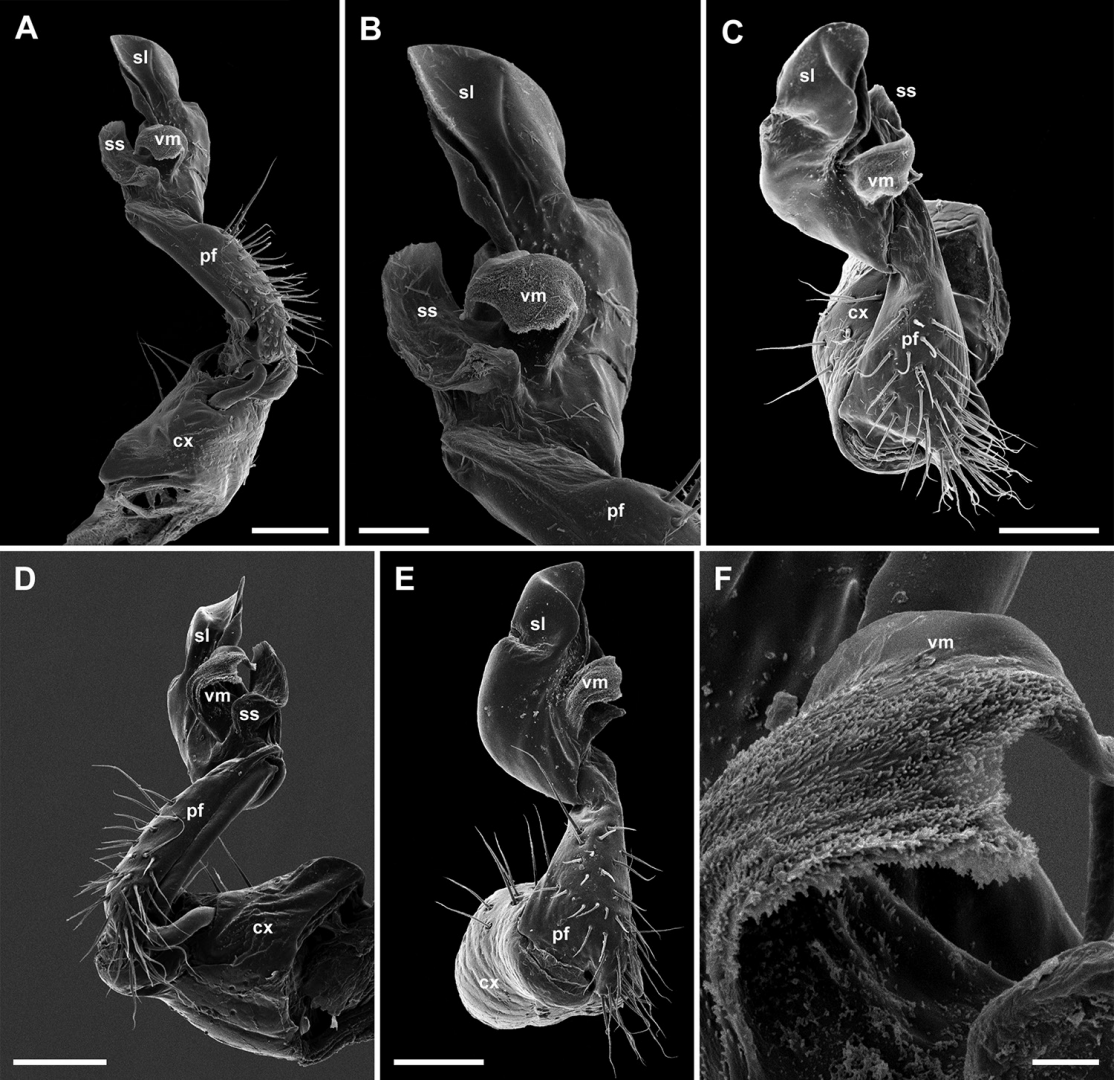
*Metonomastuspetrovi* sp. n. **A** paratype ♂, left gonopod, ventromesal view **B** paratype ♂, distal part of left gonopod, ventromesal view **C** paratype ♂, right gonopod, ventral view **D** non-type ♂, right gonopod, mesal view **E** non-type ♂, right gonopod, ventral, slightly lateral view, **F** non-type ♂, right gonopod, ventromesal process, same view, enlarged. Abbreviations: **cx** coxite, **pf** prefemorite, **sl** solenomere, **ss** saddle-shaped process, **vm** ventromesal process. Scale bars: 0.2 mm (**B**), 0.05 mm (**A, C, D, E**), 0.005 mm (**F**).

Gonopods (Figs 3C, 4): Coxite (cx) massive, moderately long, dorsolaterally very sparsely setose. Prefemorite (pf) somewhat longer than acropodite, densely setose ventrally. Postfemoral sulcus distinct, short, traceable on median and, partly, ventral sides. Acropodite consisting of three branches: mesal process (ss) curved and saddle-shaped, ventromesal process (vm) more strongly arched, its outer surface microspiculate (Figure 4F), solenomere (sl) robust, blunt, somewhat beak-shaped, ventrolaterally with a deep and broad transverse groove. Seminal groove running on mesal side all along prefemorite, then shifting dorsally on acropodite, terminating ventromesally on solenomere.

#### Remarks.

This species is known both from caves and from an epigean environment. Like some other representatives of the genus, this new species can be considered a troglophile.

### Metonomastus radjai sp. n.

Taxon classificationAnimaliaPolydesmidaParadoxosomatidae

http://zoobank.org/C1C9931C-C20A-49F6-A91E-FD1E810385AD

**Figure 5.**
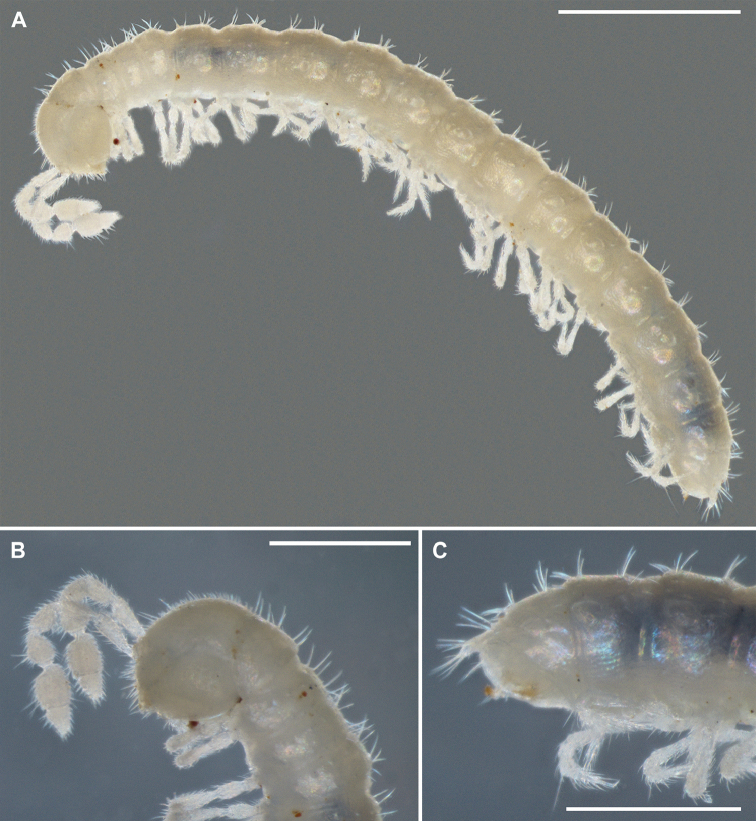
*Metonomastusradjai* sp. n., paratype ♀ (IZB) **A** habitus, lateral view **B** anterior part of the body, lateral view **C** posterior part of the body, lateral view. Scale bars: 1 mm (**A**), 0.5 mm (**B, C**).

**Figure 6.**
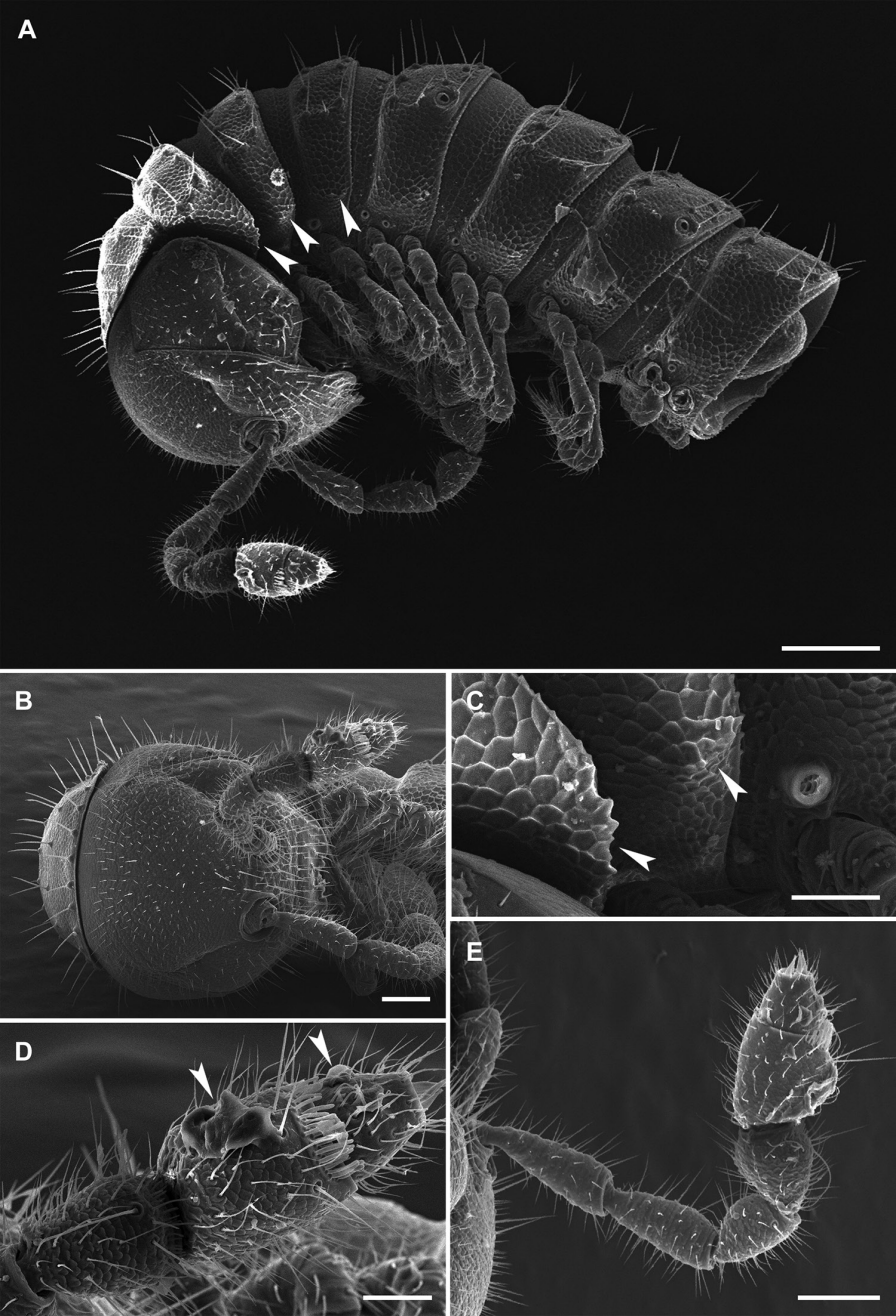
*Metonomastusradjai* sp. n., paratype ♀ (IZB) **A** anterior part of the body, lateral view **B** head, frontal view **C** pleurosternal carinae of the body rings 2 and 3, lateral view **D** tip of left antenna (with artefacts) **E** left antenna. Arrows indicate pleurosternal carinae (**A, C**) or artefacts on antenna (**D**). Scale bars: 0.2 mm (**A**), 0.1 mm (**B, E**), 0.05 mm (**C, D**).

**Figure 7.**
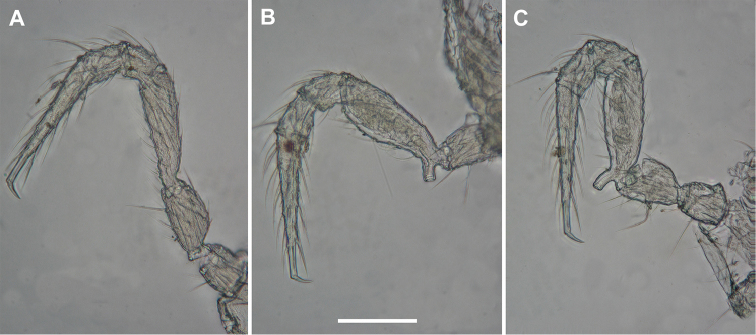
*Metonomastusradjai* sp. n., paratype ♂ (IZB) **A** leg 4 **B** leg 5 **C** leg 6. Scale bar: 0.1 mm.

**Figure 8.**
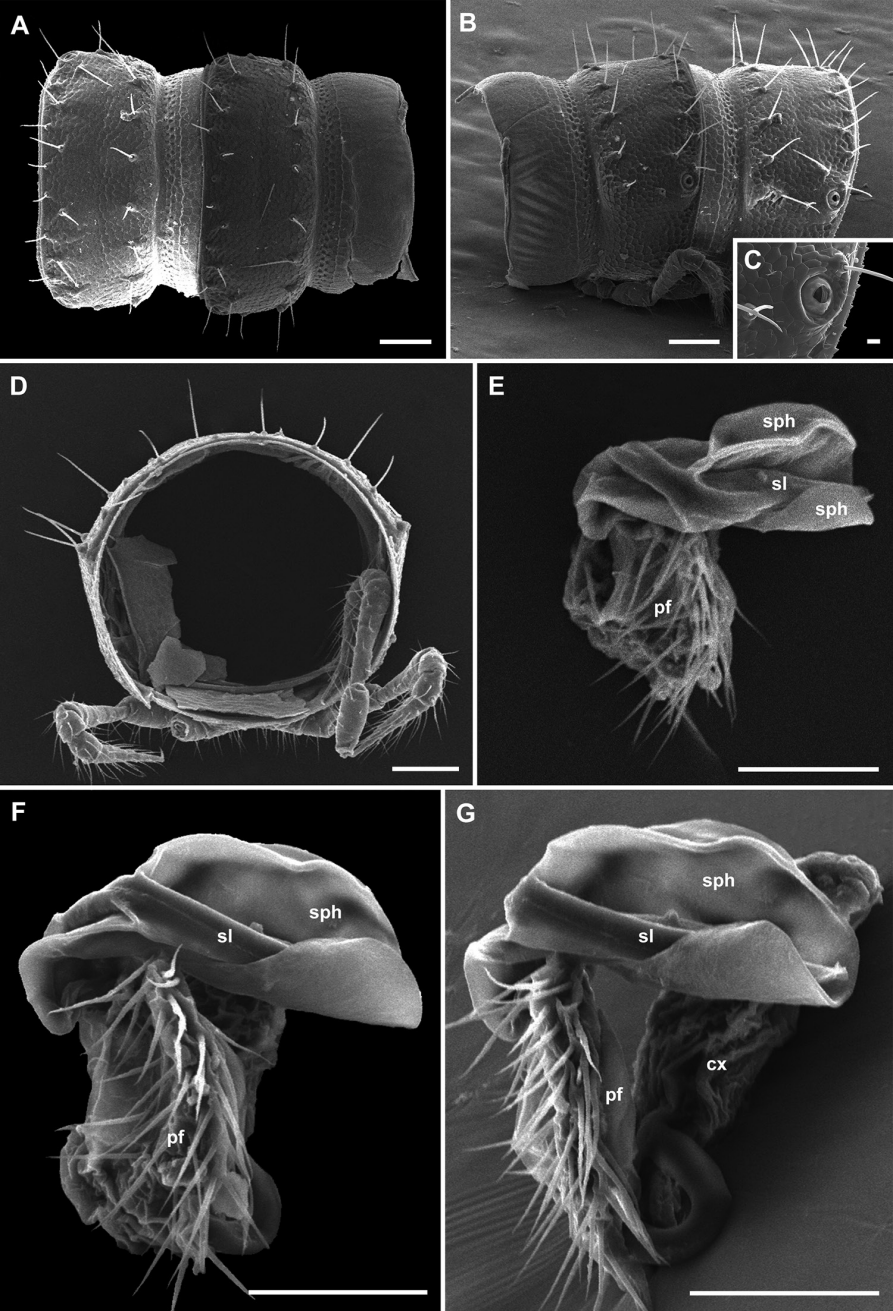
*Metonomastusradjai* sp. n., **A, B, C, D** paratype ♀ (IZB) **A** body rings 9 and 10, dorsal view **B** body rings 9 and 10, lateral view **C** body ring 10, ozopore lateral view **D** body ring 11, caudal view **E, F, G** paratype ♂ (IZB), right gonopod **E** oral (distal) view **F** ventral view **G** ventromesal view. Abbreviations: **cx** coxite, **pf** prefemorite, **sl** solenomere, **sph** solenophore. Scale bars: 0.1 mm (**A, B, D**), 0.05 mm (**E, F, G**), 0.01 mm (**C**).

**Figure 9.**
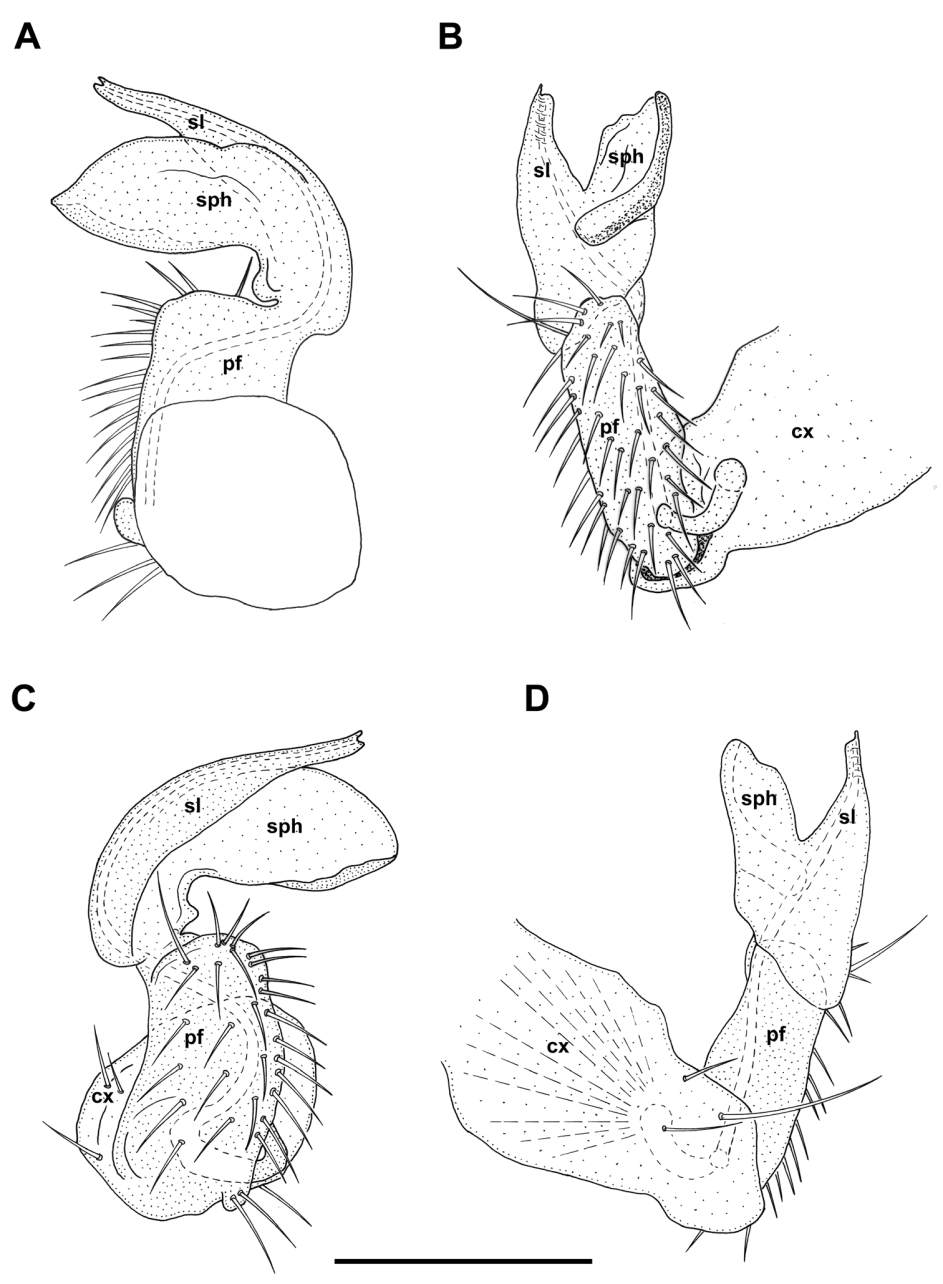
*Metonomastusradjai* sp. n. paratype ♂ (IZB), right gonopod **A** dorsal view **B** mesal view **C** ventral view **D** lateral view. Abbreviations: **cx** coxite, **pf** prefemorite, **sl** solenomere, **sph** solenophore. Scale bar: 0.1 mm.

Figs 5 , 6 , 7 , 8 , 9

#### Material examined.

**Holotype** male (NHMSC), Croatia, Dalmatia, island of Mljet, Blato, near Kozarica, under stones, 23.XII.2015, T. Rađa leg.

#### Paratypes.

1 male (NHMSC), 2 males (IZB), 4 females (NHMSC), 7 females (IZB), 4 juveniles (IZB), same data as holotype.

#### Etymology.

The new species is named after the collector, Tonći Rađa, a renowned Croatian biospeleologist who discovered numerous new or interesting arthropods. Noun in genitive case.

#### Diagnosis.

The new species belongs to the *Metonomastus* group of species with two postfemoral branches, but clearly differs from all of these by the presence of a well-developed, broad, lamellar solenophore (= tibiotarsus) directed strongly mesad, and proximally curved ventrad. The solenomere is without additional processes and is also directed strongly mesad.

#### Description.

Length 3.7–3.9 mm (males), 4.3–5 mm (females). Width of midbody rings 0.30–0.33 and 0.37–0.4 mm (males), 0.42–0.44 and 0.47–0.5 mm (females) on pro- and metazonae, respectively. Holotype male 3.7 mm long, 0.3 and 0.37 mm wide on midbody pro- and metazonae, respectively.

All other characters as in *M.petrovi* sp. n., except as follows.

Microreticulated texure more obvious (Figs 6A, 8A, B). Width of head 0.42–0.44 mm (males), 0.5–0.55 mm (females). Antennomere length 2 = 3 = 6 > 4 = 5 > 7 > 1 (Figure 6E). Postcollum metaterga each with a barely visible, mid-dorsal, transverse sulcus (Figure 8A, B). Pleurosternal carinae present on rings 2–4 in the form of small denticles, more strongly developed on 2^nd^ ring (Figure 6A, C). Legs about 1.5 times as long as midbody height in both sexes. In males, pregonopodal legs each with a distinct dorsobasal hump on prefemur; leg-pairs 5–7 each with a ventrobasal femoral adenostyle (Figure 7B, C).

Gonopods (Figs 8E-G, 9): Coxite (cx) massive, slightly elongated, laterally with three setae, one being particularly long. Prefemorite (pf) subquadrate in ventral and dorsal views, somewhat narrower in lateral and mesal views, about as long as acropodite, densely setose ventromesally. Acropodite represented by two processes: solenomere (sl) and solenophore (sph). Both solenomere and solenophore simple and directed mesad. Solenomere ventral in position, slender, acuminate, distally terminating in two small teeth. Solenophore strongly developed, dorsal in position, wide, lamellar, proximally curved ventrad. Seminal groove running through dorsomesal side of prefemorite, then shifting laterodistally through solenomere.

#### Remarks.

This species was found under stones next to a dry stone wall close to the seashore.

## Discussion

Based on gonopod characters, species of *Metonomastus* may provisionally be divided into two groups. The first group is characterized by a two-branched acropodite, while the second group includes species with three branches. Such a division is also supported biogeographically. Taxa with two acropodital branches appear in the central Mediterranean: in northeastern and central Italy and along the Adriatic coast of the western Balkans. This group contains ten species, including *Metonomastusradjai* sp. n. Among these congeners, *M.capreae* (Verhoeff, 1942), *M.hirtellus* (Silvestri, 1903), *M.mariae* (Strasser, 1965), *M.patrizii* Manfredi, 1950, and *M.romanus* (Verhoeff, 1951) are all known from central Italy [*M.hirtellus* was recently found also in Croatia (Antić et al. 2018)], also showing a similar pattern of gonopodal structure. The prefemorite seems to be somewhat elongated, at least equal in length to the acropodite, the solenomere is a well-developed and more or less erect outer branch, with an additional small process in *M.mariae* and *M.patrizii* (this could also be true for the other three species?), while the inner branch, the solenophore (= tibiotarsus) is curved mesad. On the other hand, the very common and abundant *M.albus*, known from Slovenia, Croatia and Bosnia and Herzegovina, has gonopods with a much shorter prefemorite, while the solenophore is broad, lamellar, erect, obviously more strongly developed than the solenomere, with a deep rift between the two latter structures. Although our new island species, *M.radjai* sp. n., occurs geographically much closer to the coast-inhabiting *M.albus*, it seems to show more similarities to central Italian forms, viz., a somewhat elongated prefemorite which is almost equal in length to the acropodite, the solenophore curved mesad, and a shallow rift present between the solenophore and solenomere. However, *M.radjai* sp. n. clearly differs by both acropodital branches being strongly curved mesad. In addition, the solenophore is very broad, lamellar, and with a ventrally curved proximal edge. Similarities in the gonopodal structure between *M.radjai* sp. n. and the mostly central Italian *Metonomastus* species could be evidence of a trans-Adriatic distribution of this (sub)group. Such a distribution pattern is already known not only in some millipedes, but also in such arthropods as isopods or endogean beetles (see Antić et al. 2018). Even if it has a habitus usual for the genus, the Albanian *M.petrelensis* Mauriès, Golovatch & Stoev, 1997 has gonopods with significantly different postfemoral branches from those of the above species. Both solenomere and solenophore are simple and slender, whereas the solenomere is mesal in position and spinulose distally. This species differs also from the above ones by the absence of adenostyles on the pregonopodal femora (Mauriès et al. 1997). If we consider the habitus, one species in this group is clearly distinguished from the others: *M.saetosus* (Strasser, 1960). It is characterized by very abundant and irregular metatergal pilosity, contrary to the presence of two or three transverse rows of setae in the other known *Metonomastus* species (the exception is *M.bosniensis*, reported to lack setae, but this observation requires verification). Originally, *M.saetosus* was placed in the genus *Microdesminus* Strasser, 1960, but it was synonymized under *Metonomastus* by Golovatch and Stoev (2004).

The second group within *Metonomastus* includes three species, viz. *M.pomak*, *M.strasseri*, and *M.petrovi* sp. n. All three species are characterized by the presence of three acropodital branches. Unlike the previous, central Mediterranean group, these taxa inhabit the eastern Mediterranean within Greece and northwestern Anatolia (*M.strasseri*) or the Rhodopian part of Bulgaria (*M.pomak* and *M.petrovi* sp. n.). In both previously described species, the solenophore appears as the main, long and curved branch above the solenomere which can be long and slender in *M.strasseri* or lamelliform and broad in *M.pomak*. The new species clearly differs from both of them by the presence of a strongly developed, robust and somewhat beak-shaped solenomere which appears as the main branch, while a solenophore is fully reduced.

As already stated by Golovatch and Stoev (2004), the somewhat disjunct distribution of the representatives of *Metonomastus* may be evidence of the group being ancient and relict. On the other hand, the very small bodies and a cryptic life in the soil or caves can also account for such a sporadic known distribution. Future research in the Mediterranean and southeastern Europe, including the application of various collecting techniques, will surely result in a considerable increase in the knowledge of millipede diversity, including further progress in our knowledge of the species richness and distribution of the genus *Metonomastus*.

### Key to *Metonomastus* species

**Table:** 

1	Metatergal setae absent	*** M. bosniensis ***
–	Metatergal setae present	**2**
2	Gonopods with three acropodital branches	**3**
–	Gonopods with two acropodital branches	**5**
3	Solenophore absent	***M.petrovi* sp. n.**
–	Solenophore present	**4**
4	Solenomere lamelliform and broad	*** M. pomak ***
–	Solenomere elongated and slender	*** M. strasseri ***
5	Metaterga densely setose	*** M. saetosus ***
–	Metaterga with 2–3 rows of setae	**6**
6	Gonopod prefemorite suboval and considerably shorter than acropodite	*** M. albus ***
–	Gonopod prefemorite subquadrate and about as long as acropodite	**7**
7	Acropodital branches slender and sinuate	*** M. petrelensis ***
–	Acropodital branches stouter	**8**
8	Solenomere strongly curved mesad	***M.radjai* sp. n.**
–	Solenomere not curved mesad, but erect	***M.capreae* , *M.hirtellus* , *M.mariae* , *M.patrizii* , *M.romanus***

## Supplementary Material

XML Treatment for Metonomastus

XML Treatment for Metonomastus petrovi

XML Treatment for Metonomastus radjai
